# Dietary Bacitracin Methylene Disalicylate Improves Growth Performance by Mediating the Gut Microbiota in Broilers

**DOI:** 10.3390/antibiotics11060818

**Published:** 2022-06-17

**Authors:** Jingshang Li, Yingping Xiao, Qian Fan, Hua Yang, Caimei Yang, Guolong Zhang, Shengchang Chen

**Affiliations:** 1 Key Laboratory of Plateau Mountain Animal Genetics, Breeding and Reproduction, Ministry of Education, College of Animal Science, Guizhou University, Guiyang 550025, China; jingshangligzu@126.com; 2 State Key Laboratory for Managing Biotic and Chemical Threats to the Quality and Safety of Agro-Products, Institute of Agro-Product Safety and Nutrition, Zhejiang Academy of Agricultural Sciences, Hangzhou 310021, China; xiaoyp@zaas.ac.cn (Y.X.); yanghua@mail.zaas.ac.cn (H.Y.); 3 College of Animal Science, Zhejiang A&F University, Hangzhou 310058, China; qianf0414@163.com (Q.F.); yangcaimei2012@163.com (C.Y.); 4 Department of Animal and Food Sciences, Oklahoma State University, Stillwater, OK 74078, USA; zguolon@okstate.edu

**Keywords:** bacitracin methylene disalicylate, broilers, gut microbiota, growth performance

## Abstract

The growth performance of livestock and poultry has always been a concern. However, much work is currently focused on the selection of breeds and diets to improve the growth performance of livestock and poultry. Furthermore, numerous studies have shown that the gut microbiota is closely related to the growth performance of livestock and poultry. At present, there are many reports on the impact of antibiotic intervention on the structure of gut microbiota. However, there are few reports on the influence of antibiotic intervention on the structure of intestinal microbes and the effect of this change on growth performance. Bacitracin methylene disalicylate (BMD) intervention changes the microbial structure in the caecum of broilers at different growth stages, as shown in this study. To further reveal the potential relationship between gut microbiota changes and growth performance caused by BMD intervention, correlation analysis was used for analysis. A total of 144 1-day-old male Cobb-Vantress were randomly divided into two groups. In addition to antibiotic-free starter mash diets, starter mash diets supplemented with 55 mg/kg BMD were also used, called the CON group and the BMD group, and lasted 28 days. (1) These study results showed that adding BMD to the diet had a significant effect on the growth performance of broilers. Compared with the CON group, the body weight of the BMD group increased significantly by 11.08% and 20.13% on Days 14 and 28, respectively (*p* < 0.05). Similarly, at 0–14, 14–28 and 0–28 days of age, the average daily gain of the BMD group increased significantly by 12.28%, 24.49% and 20.80%, respectively. The average daily feed intake of the BMD group increased significantly by 18.28%, 27.39% and 24.97% (*p* < 0.05). In addition, at 0–28 days of age, the feed conversion ratio increased significantly by 5.5% (*p* < 0.05). (2) Alpha diversity results show that BMD intervention has an impact on gut microbiota at different growth stages. (3) The early intervention significantly affected 7 taxa by Day 14, followed by 22 taxa by Day 28, which is similar to the results in the caecal flora. Compared with the CON group, the *Christensenellaceae R-7 group* had the highest linear discriminant analysis (LDA) score on Day 28. In addition, Pearson’s correlation analysis showed that the *Lachnospiraceae FCS020* group was significantly negatively correlated with growth performance. In general, these results indicate that dietary supplementation of BMD has an effect on broiler gut microbiota structure and growth performance. However, changes in growth performance may be caused by the gut microbiota structure.

## 1. Introduction

There are great challenges in livestock and poultry production under the background of alternative antibiotics; in particular, production performance [1,2,3], health [4,5] and other problems are the most prominent. The discovery and use of antibiotics is a major scientific discovery [6,7,8], and the importance of antibiotics to livestock and poultry production is difficult to replace. However, with long-term use and excessive addition, the body has produced drug resistance [9,10] and antibiotic residues [11,12], which also poses a great challenge to food safety. Bacitracin methylene disalicylate (BMD) is a new type of feed additive commonly found in poultry production [13,14]. The advantage of BMD is that it is not absorbed by intestinal tissues; additionally, BMD is rich in rare amino acids such as D-type amino acids and ornithine [15]. However, with increasing pH, BMD solubility increases, and BMD can be completely dissolved in the small and large intestine [16]. Therefore, BMD is an excellent feed additive and growth promoter. As a type of antibiotic, BMD has a strong effect on Gram-positive bacteria [14]. The main mechanism is to inhibit the synthesis of the bacterial cell wall by inhibiting the dephosphorylation of pyrophosphate and preventing the transport and synthesis of peptidoglycan precursors, which in turn leads to cell wall defects and an imbalance in intracellular osmotic pressure, which eventually leads to bacterial rupture and death [17,18]. Murugesan et al. used a 432-day-old Vencobb broiler as an animal model. BMD was added to the diet, and the growth performance of broilers was found to be significantly improved. BMD, as a feed drug additive, plays an important role in livestock and poultry production.

The role of the gut microbiota has attracted increasing attention, and the gut microbiota is closely related to livestock and poultry production performance [19,20,21,22], nutritional metabolism [15,23], and immune function [24,25]. Predecessors found through the technique of faecal microbiota transplantation that the growth performance of calves after faecal microbiota transplantation was significantly increased [21], which shows that the growth performance of gut microbiota is improved, but the mechanism of action is still unclear. Additionally, the research team found in the early stage that the addition of *Clostridium butyricum* to the diet can significantly improve the growth of Muscovy ducks [26], and that the gut microbiota can also improve the growth performance of broilers [27]. Therefore, the structure of the gut microbiota changes, which leads to changes in production performance.

In summary, there is a certain relationship between antibiotics, gut microbiota and livestock and poultry production. However, there are few reports on the relationship between antibiotics and intestinal microbiota and their impact on production performance. Therefore, this experiment used the Cobb broiler animal model to explore the developmental changes of gut microbiota with BMD intervention and its impact on production performance to provide some basic data for exploring the impact of new feed drug additives on livestock and poultry production.

## 2. Results

### 2.1. Effects of BMD on Growth Performance

To study whether the intervention of BMD would change the growth performance of broilers (Table 1), broilers were weighed on Days 0, 14 and 28. Compared with the CON group, the body weight (BW) of the BMD group increased significantly at 14 and 28 days (*p* < 0.05), increasing by 11.08% and 20.13%, respectively. However, there was no significant difference on Day 0 (*p* > 0.05). Similarly, the average daily gain (ADG) and average daily feed intake (ADFI) of the BMD group increased significantly at 0–14 days, 14–28 days and 0-28 days (*p* < 0.05). ADG increased by 12.28%, 24.49% and 20.80%, respectively, and ADFI increased by 18.28%, 27.39% and 24.97%, respectively. In addition, the feed conversion ratio (FCR) increased significantly only at 0–28 days of age, increasing by 5.5%, and there was no significant difference between the other two stages (*p* > 0.05).

### 2.2. Effects of BMD on the Diversity and Structural Characteristics of Gut Microbiota in the Cecum

**Figure 1:**
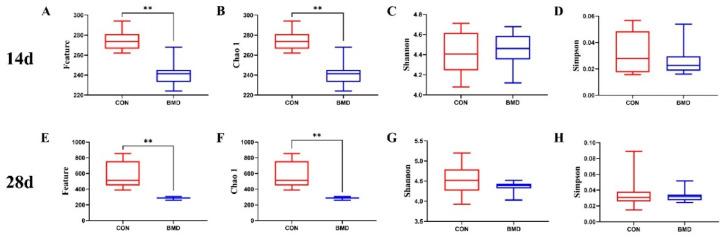
The α-diversity of the gut microbiota in the caecum of broilers in the CON and BMD groups. Features (**A**,**E**), Chao 1 (**B**,**F**), Shannon (**C**,**G**), and Simpson (**D**,**H**) were determined to elucidate the diversity and richness of the microbial community in the caecum of the broilers in the CON and BMD groups. In addition, (**A**–**D**) represent 14 days (n = 24, CON = 12, BMD = 12), and (**E**–**H**) represent 28 days (n = 23, CON = 12, BMD = 11). A two-tailed unpaired Student’s *t*-test was used to analysis the data, ** *p* < 0.01.

To investigate whether BMD intervention would change the diversity of gut microbiota in broilers, we collected the caecal contents of broilers on Days 14 and 28 and isolated DNA and sequenced the 16S rRNA gene. As shown in Figure 1, compared with the CON group, BMD intervention significantly reduced the total number of species of caecal microbiota on Days 14 and 28 (*p* < 0.01, Figure 1A,B,E,F) but had no significant effect on its species diversity (*p* > 0.05, Figure 1C,D,G,H).

**Figure 2:**
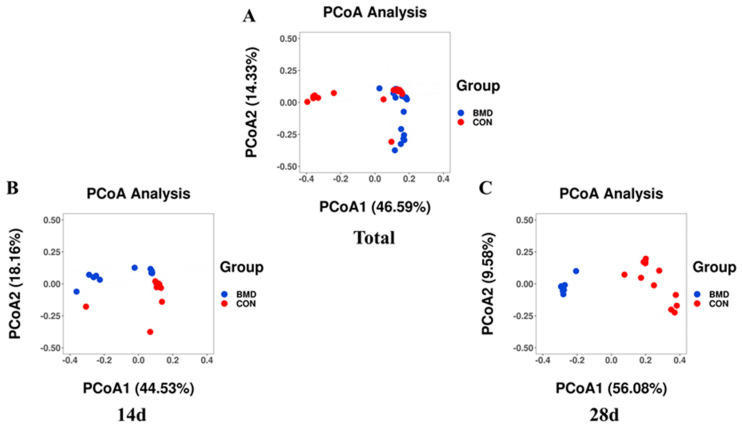
PCoA of the gut microbiota in the caecum on Day 14 (n = 24) and Day 28 (n = 23) in broilers. The caecal content of newly hatched broilers was measured on Days 14 and 28 after they were fed an unmedicated basal diet. An individual sample is represented by each data point. Based on 47 samples collected for each sampling day, PCoA was calculated (**A**,**B**) was calculated from 24 samples on each sampling day using relative abundance, and (**C**) was calculated from 23 samples on each sampling day using relative abundance. Statistical significance was determined using analysis of similarities (ANOSIM) and indicated in each plot.

In addition, to compare the differences in the microbial communities between the CON and BMD groups, we performed a Bray–Curtis differential principal component analysis (PCoA) on the β diversity of the caecal flora. The intestinal flora of the CON group and the BMD group showed a clear trend of separation, and there was a separation phenomenon in the samples in the group (Figure 2A). Therefore, on the basis of grouping, a distinction was made by age. The 14-day-old CON group had a clear separation trend from BMD, but it was not obvious (Figure 2B). Until the 28-day age range (Figure 2C), there was a clear separation trend between the two groups, indicating that there were differences in caecal microbes between the two groups at different time periods.

### 2.3. Effects of BMD on Gut Microbiota in the Cecum

**Figure 3:**
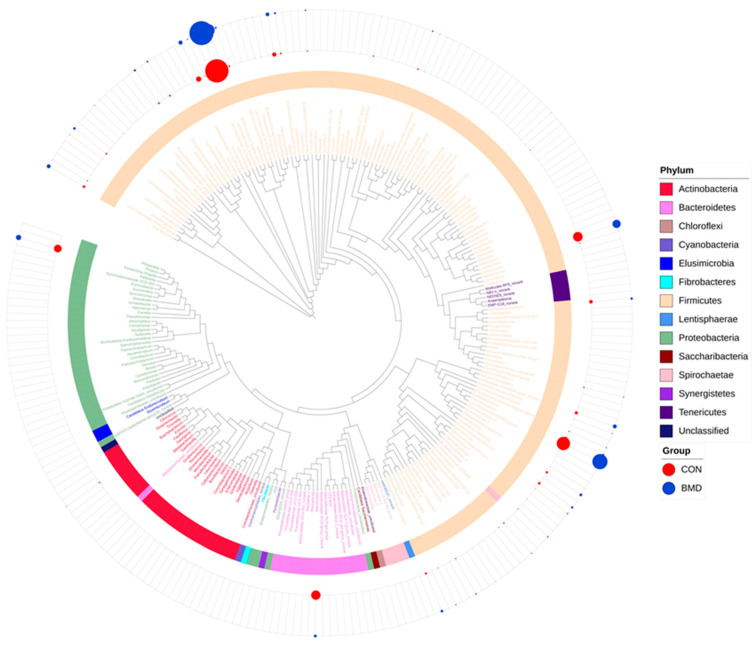
In the caecum of broilers, we found phylogenetic trees of the common bacteria. In the phylogenetic tree, the shape plot on the outermost circle was made according to the T-score; the dark orange and green solid circles represent the high and low groups, respectively; the size of the dot was determined by the value of the T-score; and the coloured strips and labels were coloured according to the phylum.

A total of 2,547,635 high-quality reads were produced by sequencing results, with an average of 54,205 reads in each sample, and were assigned to 2315 bacterial features based on 100% sequence similarity. These features were assigned to 14 phyla, 23 classes, 45 orders, 90 families, 250 genera and 478 species. For preliminary characterization of changes in bacterial flora structure, the phylogenetic tree of 47 samples was analysed based on the phylum and genus levels. The results showed that there were 14 phyla and 250 genera. *Firmicutes*, *Proteobacteria*, *Bacteroidetes* and *Actinobacteria* dominated (Figure 3).

**Figure 4:**
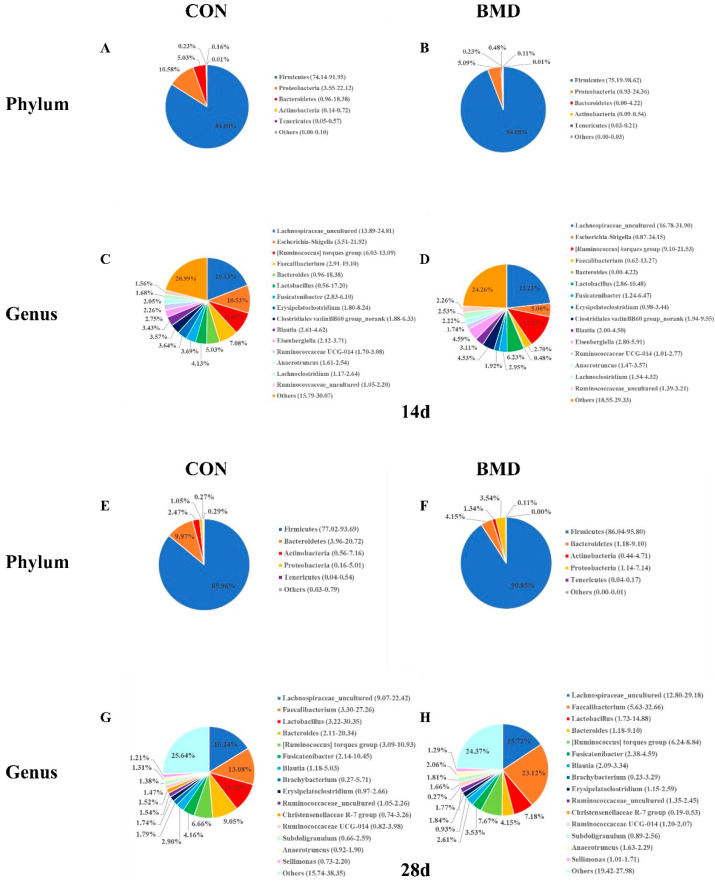
The bacterial community composition in the caecum of Day 14 (**A**–**D**) and 28 (**E**–**H**) chickens. Newly hatched chicks were fed an unmedicated basal diet for 28 days before the luminal content was collected. The composition of the bacterial community under the intervention of antibiotics was determined based on the phylum and genus levels. Only the top 5 (phyla) and top 15 (genera) are shown.

To further understand the influence of BMD intervention on the caecal microbial composition of broilers at different ages, we analysed the relative abundance of the caecal microbiota at the phylum and genus levels at 14 d and 28 d. At the phylum level (Figure 4A,B,E,F), we chose the TOP5 phylum, and the rest were defined as “others”. The results of the test showed that the types of microorganisms in the caecum of broilers were the same at the phylum level between the CON group and the BMD group, *Firmicutes*, *Proteobacteria*, *Bacteroidetes*, *Actinobacteria* and *Tenerictes*. However, their relative abundance was quite different. At the 14-day-old stage (Figure 4A,B), compared with the CON group, the BMD group *Firmicutes* was significantly increased by 10.08% (*p* < 0.05), and the *Firmicutes* level became stable at 28 days of age (Figure 4E,F), which was not statistically significant (*p* > 0.05). At the genus level (Figure 4C,D,G,H), we chose the TOP15 genus, and the rest were defined as “others”. At the 14-day-old stage (Figure 4C,D), the 15 genera of bacteria were *Lachnospiraceae*_uncultured, *Escherichia-Shigella*, *[Ruminococcus]* torque group, *Faecalibacterium*, *Bacteroides*, *Lactobacillus*, *Fusicatenibacter*, *Erysipelatoclostridium*, *Clostridiales vadinBB60 group_norank*, *Blautia*, *Eisenbergiella*, *Ruminococcaceae UCG-014*, *Anaerotruncus*, *Lachnoclostridium*, *and Ruminococcaceae*_uncultured, respectively, compared with the CON group, and we observed an increase in beneficial bacteria in the BMD group. For example, the *Lachnospiraceae*_uncultured and *[Ruminococcus]* torques groups increased by 4.1% and 3.76%, respectively. Harmful bacteria, such as *Escherichia-Shigella*, were reduced by 5.53%. At the 28-day-old stage (Figure 4G,H), the 15 species of bacteria were *Lachnospiraceae*_uncultured, *Faecalibacterium*, *Lactobacillus*, *Bacteroides*, *[Ruminococcus]* torque group, *Fusicatenibacter*, *Blautia*, *Brachybacterium*, *Erysipelatoclostridium*, *Ruminococcaceae*_uncultured, *Christensenellaceae R-7 group*, *Ruminococcaceae UCG-014*, *Subdoligranulum*, *Anaerotruncus*, and *Sellimonas*, respectively, compared with the CON group. We observed that the beneficial bacteria in the BMD group tended to be stable, such as *Lachnospiraceae*_uncultured and *[Ruminococcus]* torque group. However, *Faecalibacterium* increased significantly, increasing by 10.04%. Therefore, these findings are all related to the intervention of BMD.

**Figure 5:**
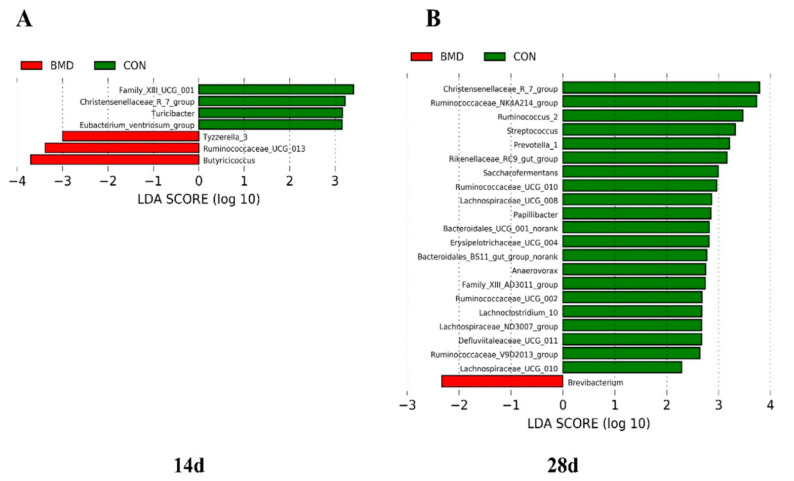
On Days 14 (**A**) and 28 (**B**) are plotted. At different growth stages, the BMD group and the CON group had different abundances of bacteria. Histograms of linear discriminant analysis (LDA) scores (threshold ≥ 2).

LefSe analysis further confirmed the difference in the relative abundance of the gut microbiota in the caecum in the CON group and the BMD group at different ages, highlighting the statistical significance and further proving the biological consistency of the intestinal flora. The logarithmic LDA score is 2.0 as a cut-off point, on Day 14, early intervention significantly impacted 7 taxa (Figure 5A), followed by 22 taxa on Day 28 (Figure 5B). Similar to the results of the caecal flora composition, the *Christensenellaceae R-7* group had the highest LDA score on the 28th day, relative to the BMD group.

### 2.4. Genus-Level Core Gut Microbiota in the Caecum of Broilers 

**Figure 6:**
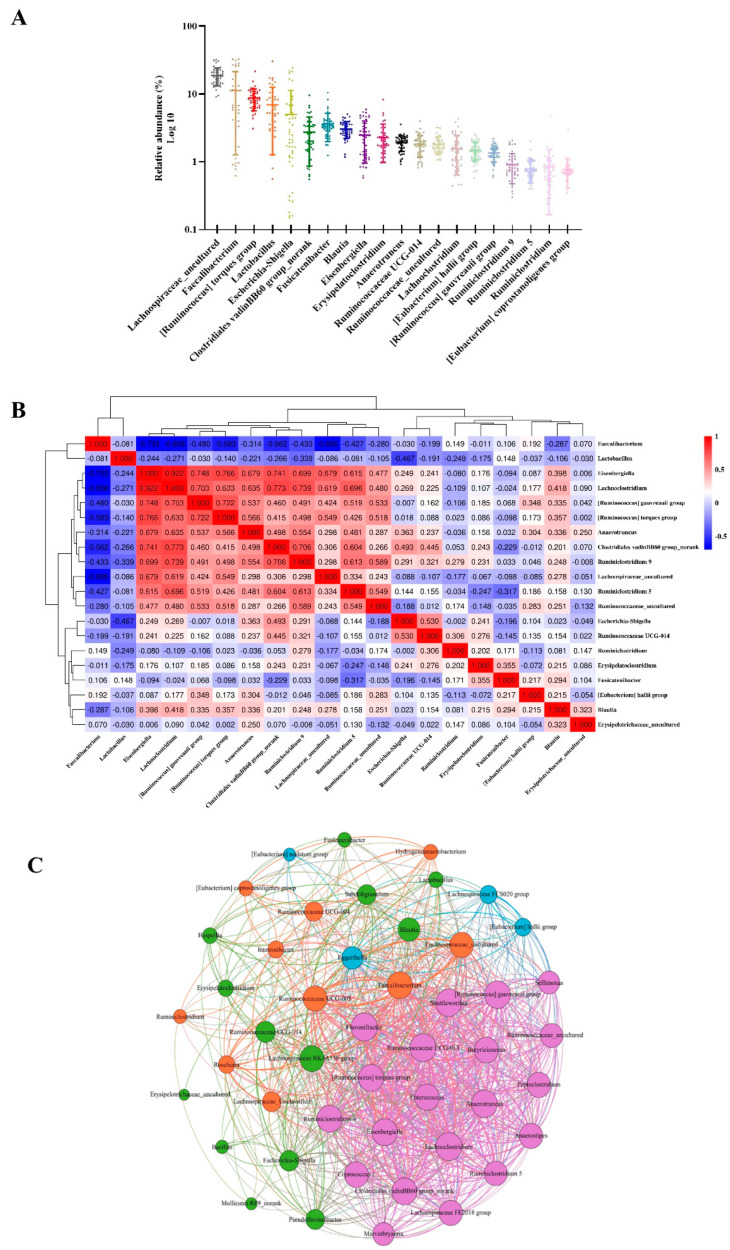
The core intestinal microbiota composed of 20 bacterial genera in broilers. (**A**) The abundance distribution of the 20 core genera. (**B**) Correlation matrix showing the Spearman’s rank correlations among the collective core, which range from −1 to 1, corresponding to a strongly positive to a strongly negative correlation, respectively. (**C**) According to the co-occurrence network, the sold line indicated that Spearman’s rank correlation coefficient was >0.5 with a *p*-value < 0.05. The size of the node was related to the abundance of the genus. Colours of nodes and lines corresponded to phylum to which the genus belongs.

To identify whether a common core microbiota is shared among all broilers, we further analysed the most abundant genera determined in this study and found 47 predominant genera shared by all sampled individuals. Then, we selected the top 20 as the core genus of broilers and used its relative abundance to make a distribution map of the core gut microbiota (Figure 6A). Excluding *Escherichia-Shigella*, which belongs to *Proteobacteria*, the other 19 species belong to *Firmicutes*. According to the Spearman rank correlation method, the cooccurrence pattern between these genera was determined (Figure 6B). *Clostridiales vadinBB60 group_norank* has a strong positive correlation with *Lachnoclostridium* (Spearman’s rank correlation coefficient (ρ) is 0.773), and *Faecalibacterium* has a negative correlation with almost all genera (Spearman’s rank correlation coefficient (ρ) is 0.192~0.733). *Eisenbergiella* and *Lachnoclostridium* have the strongest positive correlation (Spearman rank correlation coefficient (ρ) = 0.922). To reveal the interaction between the core gut microbiota in the caecum of broilers, we used the number of core bacteria in the caecum sample to generate a symbiotic network diagram based on the Spearman correlation between representative bacteria in the caecum (Figure 6C). The results showed that the gut microbiota interacted closely.

### 2.5. Correlation between the Microbiota Community and Its Growth Performance

**Figure 7:**
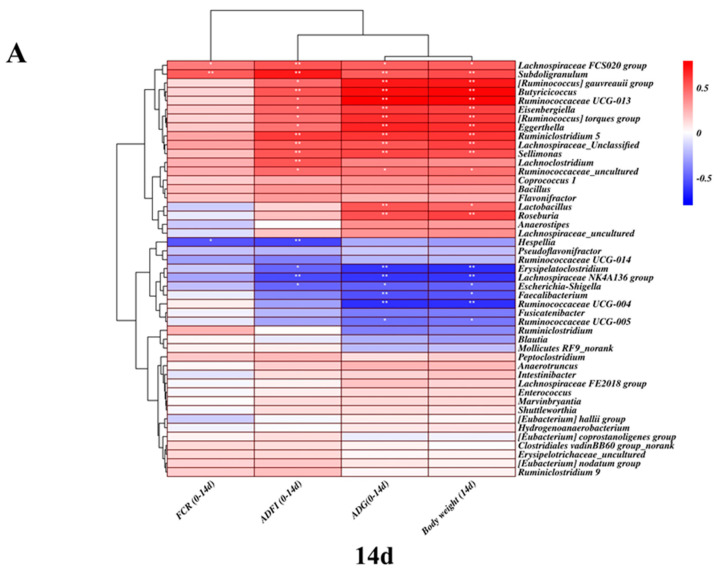

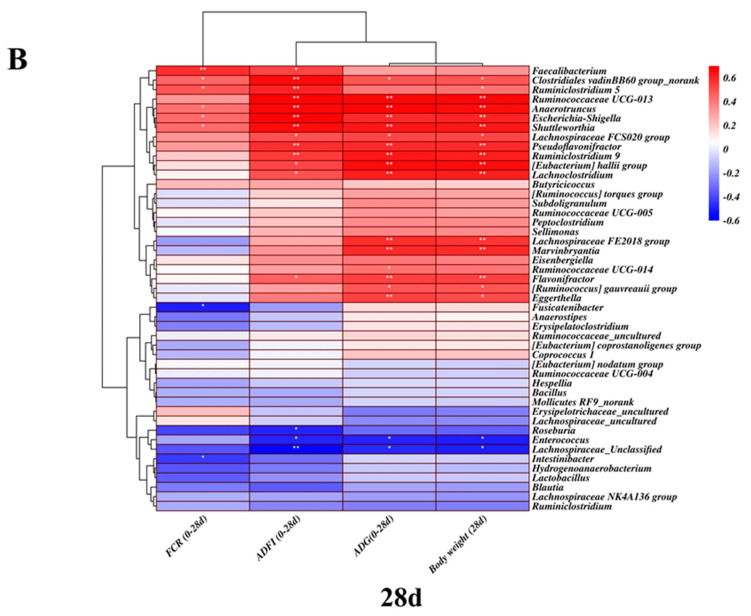
Correlation analysis between the relative abundance of different bacterial genera and the growth performance of chickens at different growth stages: (**A**) Day 14 and (**B**) Day 28. The asterisk represents its significance. * *p* < 0.05, ** *p* < 0.01.

Based on core gut microbiota, through Pearson’s correlation analysis, we found a correlation between the core gut microbiota and phenotype at different growth stages (Figure 7A,B). As shown in Figure 7A, at 14 days of age, the *Butyricicoccus*, *Ruminococcaceae 5* and *Lachnospiraceae FCS020* groups were significantly positively correlated with body weight, ADG and ADFI (*p* < 0.01). However, *Erysipelatoclostridium* and *Escherichia-Shigella* were negatively correlated with body weight, ADG and ADFI (*p* < 0.05). In addition, only the *Lachnospiraceae FCS020* group and *Subdoligranulum* were positively correlated with the false coverage rate (FCR) (*p* < 0.05), while Hespellia was significantly negatively correlated with FCR (*p* < 0.05). As shown in Figure 7B, at 28 days of age, *Lachnospiraceae FCS020* group, *Ruminococcaceae 5* and *Ruminococcaceae 9* were extremely significantly positively correlated with body weight, ADG and ADFI. Additionally, *Enterococcus* was negatively correlated with body weight, ADG and ADFI (*p* < 0.05). The *Lachnospiraceae FCS020* group was significantly positively correlated with the FCR (*p* < 0.05), and *Fusicatenibacter* was significantly negatively correlated with the FCR (*p* < 0.05).

## 3. Discussion

The performance of livestock and poultry has always been a key concern [19,21,28]. The production performance of livestock and poultry is directly affected [29,30,31]. BMD is a common growth promoter closely related to the growth performance of poultry [15,19,20,32]. Manafi et al. (2017) found that adding BMD to the diet can significantly improve the growth performance of poultry [20]. In recent years, intestinal microbes have often been mentioned, and research on intestinal microbes and the growth performance of livestock and poultry has gradually been conducted [33,34,35]. Studies by Xiao et al. (2021) found that *Clostridium butyricum* can improve Muscovy duck growth performance [26]. In addition, Gong et al. (2019) also mentioned that *Bacteroides* can improve the growth performance of poultry [27], showing that intestinal microbes also have a growth-promoting effect. The results of this experiment found that ADG, ADFI and FCR were all improved, consistent with the results of previous studies. BMD may interfere with intestinal microbes, thereby regulating the growth performance of poultry.

The purpose of this study was to explore how BMD can improve the growth performance of broilers by interfering with intestinal microbes. The diversity of Alpha and Beta shows that there are differences in the microbial structure of the caecum of broilers after BMD intervention. The alpha diversity results showed that compared with the CON group, the total number of broiler species (Feature and Chao 1) was statistically significant and the species diversity (Shannon and Simpson) was not statistically significant. We speculate that this may be because BMD intervention is extremely related to the effect of Gram-positive bacteria [14] but has no effect on the overall species diversity. Xiao et al. (2021) found that *Clostridium butyricum* intervention in Muscovy ducks at different growth stages can change the structure of intestinal flora [26]. Proctor et al. (2019) found that BMD intervention can change the microbial structure in the colon and caecum [14], and Adewole et al. (2021) found that BMD intervention can change the microbial structure in the caecum [36]. In this study, the beta diversity results showed that BMD has different effects on the caecal microbes of broilers at different ages. BMD intervention can change the microbial structure of the caecum at different growth stages, thereby affecting growth performance.

The gastrointestinal tract of poultry is closely related to the health of the host, especially the composition of the caecal microbiota [37]. To further explore the impact of BMD intervention on gut microbes, using high throughput 16S rRNA sequencing technology, our team evaluated the high-quality sequence reads obtained from 16S rRNA samples and found, on average, 54,205 high-quality sequence reads per sample, far exceeding previous studies [38,39]. First, we conducted a phylogenetic tree analysis on the microbial structure of the caecum of broilers and found that these sequences were assigned to 14 phyla, 23 classes, 45 orders, 90 families, 250 genera and 478 species, which is similar to the results of previous studies [40,41]. The four most abundant phyla were *Firmicutes*, *Proteobacteria*, *Bacteroidetes* and *Actinobacteria*, which is consistent with previous research results [40,42]. Based on the phylum level, we performed a pie chart characterization of the intestinal microbial structure and found that *Firmicutes*, *Proteobacteria*, *Bacteroidetes*, *Actinobacteria* and *Teneritictes* were the most abundant and were defined as the dominant phyla. In addition, during the initial stage of BMD intervention, the relative abundance of *Firmicutes* was increased by 10.08% compared with the relative abundance of *Firmicutes* in the CON group, which indicated that BMD intervention had a significant impact on *Firmicutes* (*p* < 0.05), consistent with the results of previous studies [43,44,45]. However, in the late stage of BMD intervention, it had no significant effect on the caecal microbes (*p* > 0.05), possibly as a result of the long-term intervention and adaptation of broilers. In the same way, the bacteria were characterised based on the genus level. The test results found that compared with the CON group, BMD intervention was not statistically significant in the early stage of the test. In the later stage of the test, *Faecalibacterium* significantly increased by 10.04%, and *Christensenellaceae R-7* significantly decreased by 1.25%. The *Faecalibacterium* and *Christensenellaceae R-7* groups belong to *Firmicutes*. Therefore, the results of this experiment are consistent with the conclusions drawn at the gate level. To further explore the impact of BMD intervention on intestinal microbes, based on LefSe analysis, other representative bacterial genera were identified as potential target bacterial genera. For example, predecessors speculated that *Christensenellaceae R-7* group may be related to glucose metabolism and amino acid metabolism [46]. In this study, the *Christensenellaceae R-7* group was significantly lower than the CON group, showing that the *Christensenellaceae R-7 group* may regulate growth performance by affecting sugar metabolism and amino acid metabolism. In summary, BMD intervention may be through intervention in *Firmicutes* to regulate growth performance, and the potential target bacteria are most likely the *Christensenellaceae R-7* group.

In terms of the core microbiome, 47 genera were detected in all broiler samples, and the top 20 were selected as the broiler caecal core microbiome. The results show that they are from *Firmicutes* and *Proteobacteria*, consistent with the results of phylogenetic tree analysis. In addition, 19 of the core microbiomes belong to *Firmicutes*, and only one belongs to *Proteobacteria*, indicating that Firmicutes has an absolute advantage in the core bacteria, consistent with the results of Oakley et al. (2014) [43]. Therefore, we speculate that it is possible to regulate growth performance and improve livestock and poultry growth performance through intervention in *Firmicutes*.

Through Pearson correlation analysis, the relative abundance of the genus was found to be closely related to growth performance. For example, the *Lachnospiraceae FCS020 group* had a very significant positive correlation with ADG and ADFI, while *Erysipelatoclostridium* had a very significant negative correlation. The *Lachnospiraceae FCS020* group is considered to be a producer of short-chain fatty acids, which are closely related to production performance [47]. However, *Erysipelatoclostridium* is a genus of bacteria that can cause inflammation in the body [48], similar to the results of this study. *Butyricicoccus* is considered to be related to necrotic enteritis [49] and regulates inflammatory response by activating short chain fatty acid (SCFA) transporters or receptors by itself or by metabolites [50]. Therefore, in general, these bacterial genera are considered potential target bacterial genera for potentially regulating the growth performance of broilers.

## 4. Materials and Methods

### 4.1. Animal Trials and Sample Collection

Animal trials and sample collection were performed as described in our previous report [51]. Briefly, all animal trials were conducted in accordance with the Institutional Animal Care and Use Committee of Oklahoma State University under protocol number AG-173. Day-of-hatch male Cobb broiler chicks were obtained from Cobb-Vantress Hatchery (Siloam Springs, AR) and randomly assigned to one of two dietary treatments with 12 birds per cage and 6 cages per treatment. Birds were provided ad libitum access to starter feed and tap water for the entire duration of the trial. Dietary treatments included an antibiotic-free standard corn-soybean starter mash diet or a starter mash diet supplemented with BMD at subtherapeutic (55 mg/kg) levels for up to 28 days. Birds were raised in floor cages with fresh dry pine wood shavings under standard management in an environmentally controlled room with temperatures starting at 33 °C and decreasing 3 °C every 7 days. Lighting for this trial included a light-to-dark ratio of 23:1 from Days 0 to 7, 16:8 from Days 8 to 22, 17:7 on Day 23, and 18:6 from Days 24 to 28. To minimise cross-contamination, birds in different treatments were housed in separate rows with physical barriers between. On Days 14 and 28, 2 birds per cage and 12 birds per treatment were euthanised via CO_2_ asphyxiation, and the caecal contents were collected to evaluate the effect of BMD on the caecal flora. The samples were immediately frozen in liquid nitrogen and stored at −80 °C until further processing.

### 4.2. DNA Extraction and Sequencing

Genomic DNA was extracted from each caecum sample using a QIAamp DNA Stool Mini Kit (Qiagen, Valencia, CA, USA) according to the instructions. The 1% agarose gel electrophoresis and NanoDrop ND-1000 (Thermo Fisher Scientific, Waltham, MA, USA) were used to evaluate the quality and concentration of DNA extracts, and then high-quality DNA was used for sequencing. In detail, the V3-V4 region of the bacterial 16S rRNA gene was amplified using the barcode-fusion forward primer 341F (5′-CCTAYGGGRBGCASCAG-3′) and the reverse primer 806R (5′-GGACTACNNGGGTATCTAAT-3′). The polymerase chain reaction (PCR) conditions were as previously described [52]. The reaction was carried out using Phusion High-Fidelity PCR Master Mix (New England Biolabs) under the following conditions: initial denaturation at 98 °C for 1 min, followed by 30 cycles of denaturation at 98 °C for 10 s, annealing at 50 °C for 30 s, elongation at 72 °C for 30 s, and a final extension at 72 °C for 5 min.

After PCR, a 2% (*w*/*v*) agarose gel was used to separate and qualify the amplicons, which were then purified by a GeneJET Gel Extraction Kit (Thermo Scientific, Waltham, MA, USA) according to the manufacturer’s instructions. An Illumina TruSeq DNA PCR-Free Library Preparation Kit (Illumina) was applied for sequencing library generation. A Qubit 2.0 Fluorometer (Thermo Scientific) and an Agilent Bioanalyzer 2100 system were used to evaluate the quality of the generated library. The qualified library was sequenced commercially by Novogene on an Illumina NovaSeq platform, generating 466 bp paired end reads. 

### 4.3. Bioinformatics Analysis

Bioinformatics analysis was performed as described in our previous report [51]. Briefly, Illumina paired end reads were analysed using the deblur program in QIIME 2 (version 2019.7). Deblur is capable of achieving single-nucleotide resolution based on error profiles within samples, and it produces denoised sequences known as amplicon sequence variants (ASVs) or exact sequence variants (ESVs), which can be compared between studies. Bacterial sequences were processed using the “deblur denoise-other” option with positive alignment-based filtering against the UNITE reference database. Denoised sequences were classified into bacterial features using the Warcup V4-V5 database and the Ribosomal Database Projects (RDP) Bayesian classifier. A bootstrap confidence of 80% was used for taxonomic classification. Features with a classification of less than 80% were assigned the name of the last confidently assigned level followed by “_unidentified”. Species classification of the top 20 operational taxonomic units (OTUs) was confirmed through a BLASTN search of the nucleotide database of GenBank. Features appearing in less than 5% of samples were removed from downstream analysis. Data were normalised using cumulative sum scaling in the metagenomeSeq package of R. Analysis and visualisation of the microbiota composition were conducted in R version 3.5.1. The α- and β-diversities were calculated with the phyloseq package version 1.24.2. Plots were made using ggplot2 version 3.0.0.

α-diversity (Feature, Chao 1 index, Shannon index, and Simpson index) and beta-diversity were calculated and visualised in Origin Lab 2018 (Northampton, MA, USA). In the Interactive Tree of Life, the rooted phylogenetic tree was visualised (iTOL, https://itol.embl.de/; version 5.5, accessed on 1 January 2022). Spearman correlation analysis was performed to identify a possible correlation between individual features and BMD inclusion using the Benjamini–Hochberg correction. Features were considered significant if the *p* value was ≤0.05. The raw 16S rRNA gene sequencing data are available in the SRA database under Accession Number PRJNA512838.

### 4.4. Statistical Analysis

OriginLab 2018 was used for all statistical analyses. Data are expressed as the mean. Unpaired two-tailed Student’s *t*-test was used to analyse the difference between groups, and the *p* value was considered significant when it was no greater than 0.05. To generate phylogenetic trees, the T-score was calculated based on the average abundance of the common bacterial genera present in 50% of the caecum samples in the CON and BMD groups according to the following formula [53]:

$$T=50+10\times \left(\frac{X-\bar{X}}{s}\right)$$

where *X* denotes the original data, $\bar{X}$ denotes the average, and *S* denotes the standard deviation.

The top 20 genera with the highest relative abundance were selected for core flora analysis.

## 5. Conclusions

In summary, BMD promotes growth by regulating the intestinal microbes of broilers. There are two main ways. First, *Butyricicoccus* activates SCFA transporters, receptors, or their metabolites to regulate inflammation and achieve the purpose of promoting growth. Second, the *Lachnospiraceae FCS020* group promotes growth by regulating sugar metabolism and amino acid metabolism. Therefore, we speculate that BMD promotes growth performance by regulating gut microbiota.

## Figures and Tables

**Table 1:** The growth performance of broilers in the CON and BMD groups.

Item	CON	BMD	SEM	*p*-Value
Body weight (g)				
Day 0	45.58	45.81	0.36	0.755
Day 14	393.57	442.59	5.32	<0.001
Day 28	1088.03	1362.18	29.71	<0.001
Average daily gain (g)				
0–14 d	24.86	28.34	0.38	<0.001
15–28 d	49.60	65.69	1.77	<0.001
0–28 d	37.23	47.01	1.06	<0.001
Average daily feed intake (g/d)				
0–14 d	40.58	49.66	1.32	<0.001
15–28 d	99.49	137.02	4.12	<0.001
0–28 d	70.03	93.34	2.56	<0.001
Feed conversion (g/g)				
0–14 d	1.64	1.75	0.04	0.123
15–28 d	2.00	2.09	0.03	0.168
0–28 d	1.88	1.99	0.03	0.037

Note: A two-tailed unpaired Student’s *t*-test was used to analysis the data.

## Data Availability

The raw 16S rRNA gene sequencing data are available in the SRA database under Accession Number PRJNA512838.
